# The remote administration of the uniform data set neuropsychological test battery (I-UDSNB) Italian version: normative data

**DOI:** 10.1007/s10072-025-08458-3

**Published:** 2025-09-20

**Authors:** Davide Quaranta, Francesca Conca, Federica L’Abbate, Valentina Esposito, Elena Gobbi, Ilaria Pagnoni, Francesca Baglio, Francesca Borgnis, Maddalena De Matteis, Michelangelo Stanzani-Maserati, Federica Piras, Giulia Caruso, Valentina Catania, Francesco Rundo, Barbara Poletti, Vincenzo Silani, Matteo Pardini, Beatrice Orso, Emanuela Inguscio, Valeria Crepaldi, Nicola Canessa, Giulia Mattavelli, Alberto Albanese, Elena Perdixi, Andrea Pace, Antonio Tanzilli, Maria Cotelli, Raffaele Lodi, Raffaele Ferri, Pietro Tiraboschi, Fabrizio Tagliavini, Sonia Di Tella, Ugo Lucca, Alessia A. Galbussera, Mauro Tettamanti, Eleonora Catricalà, Stefano F. Cappa, Camillo Marra

**Affiliations:** 1 Memory Clinic - Neurology Unit, Fondazione Policlinico Universitario “A. Gemelli” IRCCS, L.go A. Gemelli, 8, Rome, 00168 Italy; 2 Department of Neuroscience, Catholic University of the Sacred Heart, Milan, Italy; 3 IUSS Cognitive Neuroscience (ICoN) Center, Institute for Advanced studies, Pavia, Italy; 4 IRCCS Mondino Foundation, Pavia, Italy; 5 Neuropsychology Unit, IRCCS Istituto Centro San Giovanni di Dio Fatebenefratelli, Brescia, Italy; 6 IRCCS Fondazione Don Carlo Gnocchi, ONLUS, Milan, Italy; 7 IRCCS Istituto delle Scienze Neurologiche di Bologna, Bologna, Italy; 8 Clinical Neuroscience and Neurorehabilitation Department, IRCCS Fondazione Santa Lucia, Rome, Italy; 9 Neuroimaging Laboratory, IRCCS Fondazione Santa Lucia, Rome, Italy; 10 Oasi Research Institute-IRCCS, Troina, Italy; 11 Department of Neuroscience, IRCCS Istituto Auxologico Italiano, Milan, Italy; 12 Department of Oncology and Hemato-Oncology, Università degli Studi di Milano, Milan, Italy; 13 Department of Pathophysiology and Transplantation, “Dino Ferrari” Center, Università degli Studi di Milano, Milan, Italy; 14 IRCCS Ospedale Policlinico S. Martino, Genoa, Italy; 15 Fondazione IRCCS Istituto Neurologico Carlo Besta, Milan, Italy; 16 Cognitive Neuroscience Laboratory of Pavia Institute, Istituti Clinici Scientifici Maugeri IRCCS, Pavia, Italy; 17 Department of Neurology, IRCCS Humanitas Research Hospital, Milan, Italy; 18 Neuro-Oncology Unit, I.R.C.C.S. Regina Elena National Cancer Institute, Rome, Italy; 19 Istituto di Ricerche Farmacologiche Mario Negri IRCCS, Milan, Italy

**Keywords:** Neuropsychological tests, UDS, Remote administration

## Abstract

The availability of remotely administered neuropsychological batteries is crucial to provide access to care in extraordinary situations, e.g., the recent pandemic, and for individuals with reduced mobility. Here we present the normative data of the remotely administered version of the Italian Uniform Data Set Neuropsychological Battery (tele-I-UDSNB), developed by our group. I-UDSNB included Craft Story, Benson Figure, Digit Span, Semantic and Phonemic Fluency, Trail Making Test A and B, Picture Naming, and the Five Words Test, which were adapted to be administered via web-based communication software. The tele-I-UDSNB was administered to 157 healthy participants who also underwent the face-to-face version of the battery. Regression models were used to evaluate the impact of demographic variables on performance and to obtain reference norms. The effect of modality and order of administration was assessed by factorial ANOVAs. Age predicted the performances on most of the tests, whereas education was associated with performance on Craft Story, Benson Figure, Digit Span, Semantic and Phonemic Fluency, and Trail Making Test. Sex affected some subscores of Semantic Fluency and Digit Span. The modality of administration showed little influence on the performance, limited to scores related to Semantic Fluency. The tele-I-UDSNB could be a useful tool for tele-neuropsychological assessment, with the modality of administration only showing a limited effect on some sub-scores.

**Supplementary Information:**

The online version contains supplementary material available at 10.1007/s10072-025-08458-3.

## Introduction

Neuropsychological assessment maintains crucial importance to define the presence of objective cognitive impairment required for the diagnosis of prodromal Alzheimer’s disease (AD) [1]. In standard clinical practice, it represents the first element motivating further biomarkers investigation [2]. Several initiatives aimed at developing harmonized sets of neuropsychological tests, overcoming the variability among clinical centers and providing high quality normative data. In particular, the Uniform Data Set – Neuropsychological Battery (UDSNB) was developed by the Alzheimer’s National Coordinating Center for this purpose and has been successively updated to the most recent version, i.e., UDSNB 3.0 [3]. Following the recommendations of a European consensus conference [4] our working group has recently developed an Italian tablet-based version of the UDSNB (I-UDSNB), that was standardized on 433 healthy subjects [5]. The clinical validity of the I-UDSNB has been reported for the diagnosis of Mild Cognitive Impairment and early AD [6].

In the last few years, a further need, namely the possibility of administering neuropsychological assessment remotely, emerged significantly. The assessment of patients who are not physically present for the traditional face-to-face examination may be an opportunity to warrant equal access to care for people with reduced mobility or other special needs. Although this kind of examination has been tested for at least a decade in different populations, including healthy participants and patients affected by psychiatric or neurodegenerative diseases [7, 8], the recent pandemic gave a significant impulse to the development and validation of remotely administered neuropsychological tests [9]. The currently available evidence supports the feasibility and reliability of this approach, often based on the adaptation of traditional face-to-face tests to remote administration [10]. More specifically, there is evidence that tele-neuropsychology may be reliable for screening purposes and for the evaluation of AD in prodromal and mild to moderate stages [11].

The UDSNB 3.0 has been recently normed and validated for remote administration [12]. The aim of the present project is the development and standardization of a remotely administered version of the I-UDSNB (tele-I-UDSNB).

## Methods

### Sample

The sample was composed of 157 cognitively healthy individuals (92 females and 65 males) who had been previously enrolled for the collection of normative data of the face-to-face version of the I-UDSNB [5]. The mean age of participants was 60.1 years (SD = 12.3; range = 40–89), and the mean years of formal education was 13.5 years (SD = 4.15; range = 5–18) (see Table 1). Participants were excluded in the case of: prior/current neurological or major psychiatric disorders; history of traumatic brain injury, brain tumors, or stroke; history of alcohol or drug abuse; age- and education-corrected score ≤ 24 on the Mini-Mental State Examination; sensory or motor deficits possibly affecting performance; exposure to anesthesia in the previous 3 months. In the present study, a further exclusion criterion was represented by the unavailability of an internet connection and a device (desktop, laptop, or tablet) suitable for the administration of the battery. The latter criterion is acknowledged to represent a source of potential bias [13].

**Table 1:** Distribution of the sample stratified according to age, years of formal education, and sex. M = male, f = female

Education/age	40–49	50–59	60–69	70–79	80–89	Total
≤ 8	2 (1 F/1 M)	9 (5 F/4 M)	12 (8 F/4 M)	8 (7 F/1 M)	1 (0 F/1 M)	32 (21 F/11 M)
9–13	11 (6 F/5 M)	22 (15 F/7 M)	19 (10 F/9 M)	4 (2 F/2 M)	5 (4 F/1 M)	61 (34 F/24 M)
≥ 14	22 (10 F/12 M)	10 (6 F/4 M)	15 (8 F/7 M)	13 (7 F/6 M)	4 (3 F/1 M)	64 (34 F/30 M)
Total	35 (17 F/18 M)	41 (26 F/15 M)	46 (26 F/20 M)	25 (16 F/9)	10 (7 F/3 M)	157 (92 F/65 M)

In 137 individuals the battery was administered first face-to-face and then remotely (i.e., tele-I-UDSNB), while the reverse order was used in the remaining 20 participants. The mean time interval between the two administrations was 227 days (SD = 69.5).

### Procedures

The tele-I-UDSNB was adapted from the face-to-face version of the test, developed by 17 centers belonging to the Virtual Dementia Institute of the RIN group (Rete Italiana di Neuroscienze e Neuroriabilitazione-Italian Network of Neuroscience and Neuro-rehabilitation) [5]. The resulting tablet-based application allowed for collecting scores via a web-based data entry system. The battery included the following tests in order of administration: Craft Story, Benson Figure (copy and recall); Digit Span forward and backward; Semantic Fluency (animals and vegetables); Trail Making Test A and B (TMT-A, TMT-B); Picture Naming; Phonemic Fluency (F and L); Five Words Test.

The tele-I-UDSNB was administered via teleconference using the tablet application originally developed for in-person examination. Some tests were adapted for remote administration, as described here. Specifically, the Digit Span and the Semantic and Phonemic Fluency tests were administered following the same procedures as the face-to-face sessions, with participants’ responses recorded through the tablet’s microphone. For the Five Words test and Picture Naming, the examiner shared the materials via the screen-sharing feature, while participants’ answers were captured via the tablet’s microphone. In the case of the Benson Figure test, the examiner displayed the figure through screen sharing; after completing the task, participants were asked to photograph and submit their copied and recalled figures using the teleconference application. Lastly, the TMT-A and TMT-B training materials and response sheets were sent to participants in advance with instructions to print them before the evaluation; during the assessment, participants were instructed to keep their response sheets clearly visible to the camera.

For all tests, participants’ responses were scored as in the face-to-face version of the I-UDSNB [5].

### Statistical analysis

#### Normative data

The raw scores obtained on each test were set as dependent variables in univariate linear regression models in which age, education, and sex (coded as female = 1, and male = 0) were entered as predictors. Age and education were also used as predictors after transformation (logarithmic [ln(100-age); ln(30-education)], quadratic, cubic, and square root). In the I-UDSNB the two scores expressed as dichotomous values, namely the recognition of the Benson figure (i.e. 0 = incorrect recognition, 1 = correct recognition) and the use of the cue in the recall of the Craft Story (i.e. 0 = cue not required, 1 = cue required) were not analyzed following the previously adopted procedures [3, 5]. When multiple variable transformations were statistically significant, we selected the simplest form (e.g., age instead of its transformations) only if the difference in explained variance between models (expressed as R²) was less than 0.009, to be in line with the procedure previously adopted [5]. If the difference exceeded this threshold (R² >0.009), the transformation yielding the highest explained variance was included in the multiple regression models. Only predictors that were significant in univariate regressions were entered in the final multiple-variable regression models.

The multiple variable models were used to obtain corrected scores for each neuropsychological test. Thereafter, adjusted scores were ordered from the worst to the best performance and classified into five equivalent scores (ES), from 0 to 4 [14]. Cut-off points corresponded to the outer non-parametric tolerance limits that allowed to leave above at least 95% of the population, with 95% of confidence (corresponding to the 4th observation for 157 participants), and values equal or lower/higher than the cutoff value were defined as pathological and assigned an ES of 0. An ES of 4 was assigned to scores above the median corrected score. ES 1, 2, and 3 were obtained by subdividing the scores above ES = 0 (outer tolerance limit) and below ES = 4 (median) in three parts [14].

#### Modality and order of administration effects

A 2 × 2 factorial ANOVA for each test score, including MODALITY (FACE-TO-FACE vs. REMOTE administration) and ORDER (administered as FIRST vs. SECOND) as factors, was performed. Post-hoc pairwise comparisons were also computed when a significant effect of MODALITY X ORDER interaction was observed. Since the group of participants undergoing first the tele-I-UDSNB in face-to-face modality was larger than the subgroup receiving first the remote modality, an ANOVA was carried out by selecting twenty subjects of the first group that were age, education, and sex-matched to the participants in the second group. Since the time between the two administrations showed some variability, the interval was set as a covariate in the model. Correction for multiple comparisons was carried out by applying the False Discovery Rate method [15].

## Results

### Normative data

Descriptive statistics, regression equations, and cut-off values for each test are shown in Table 2.

**Table 2:** Descriptive statistics, regression equations and cut-off values for each test. - indicates that the regression equation is not computed. Of note, for the craft story we considered here only the verbatim and not the paraphrase score. PVF: phonological verbal fluency; s: seconds; SVF: semantic verbal fluency; TMT: trail making test

	Mean (SD)	Range	Equation	Cut-off
Craft story immediate verbatim	17.45 (6.79)	3–36	Corrected score = raw score - [(−0.0960)*(age − 60.07) + (−6.5731)*(ln(30 - education) − 2.76)]	≤ 7.336
Craft story recall verbatim	14.76 (6.72)	2–35	Corrected score = raw score - [(−0.1288)*(age − 60.07) + (−6.1601)*(ln(30 - education) − 2.76)]	≤ 4.207
5 words Immediate free recall	4.58 (0.65)	2–5	Corrected score = raw score - [(−0.0122)*(age − 60.07)]	≤ 2.963
5 words Immediate cued recall	0.36 (0.58)	0–2	Corrected score = raw score - [(0.0079)*(age − 60.07)]	≥ 1.874
5 words Immediate total recall	4.94 (0.24)	4–5	-	≤ 4
5 words Immediate total-weighted	9.51 (0.78)	6–10	Corrected score = raw score - [(−0.0164)*(age − 60.07)]	≤ 7.114
5 words Delayed free recall	4.38 (0.82)	2–5	Corrected score = raw score - [(−0.0183)*(age − 60.07)]	≤ 2.145
5 words Delayed cued recall	0.41 (0.59)	0–2	Corrected score = raw score - [(−0.2556)*(ln(100 - age) − 3.63)]	≥ 2.009
5 words Delayed total recall	4.80 (0.48)	3–5	-	≤ 3
5 words Delayed total-weighted	9.18 (1.21)	5–10	Corrected score = raw score - [(−0.0293)*(age − 60.07)]	≤ 6.027
5 words Total free recall	8.96 (1.25)	5–10	Corrected score = raw score - [(1.0676)*(ln(100 - age) − 3.63)]	≤ 6.049
5 words Total cued recall	0.77 (0.98)	0–4	Corrected score = raw score - [(0.0152)*(age − 60.07)]	≥ 3.153
5 words Total recall	9.73 (0.60)	7–10	Corrected score = raw score - [(−0.0152)*(age − 60.07)]	≤ 8.090
5 words Total-weighted recall	18.69 (1.70)	14–20	Corrected score = raw score - [(1.5941)*(ln(100 - age) − 3.63)]	≤ 14.418
Picture naming correct without cue	31.35 (1.56)	20–32	Corrected score = raw score - [(−0.0379)*(age − 60.07)]	≤ 28.225
Picture naming correct with cue	0.15 (0.43)	0–3	Corrected score = raw score - [(0.0091)*(age − 60.07)]	≥ 1.419
Picture naming correct total	31.50 (1.19)	23–32	Corrected score = raw score - [(−0.0279)*(age − 60.07)]	≤ 28.665
PVF F < 30 s	9.01 (3.31)	3–12	Corrected score = raw score - [(0.2060)*(education − 13.52)]	≤ 3.136
PVF F > 30 s	5.77 (2.67)	3–7	Corrected score = raw score - [(0.1405)*(education − 13.52)]	≤ 0.370
PVF F Total (60 s)	14.78 (5.20)	6–19	Corrected score = raw score - [(1.1604)*(education − 13.52) + (−0.0301)*(education^2^ − 199.80)]	≤ 5.539
Perseveration F	0.71 (1.38)	0–5	-	≥ 5
Violation F	0.22 (0.547)	0–2	-	≥ 2
PVF L < 30 s	7.55 (2.91)	2–10	Corrected score = raw score - [(0.7337)*(education − 13.52) + (−0.0190)*(education^2^ − 199.80)]	≤ 2.245
PVF L > 30 s	4.30 (2.51)	0–8	Corrected score = raw score - [(0.5817)*(education − 13.52) + (−0.0155)*(education^2^ − 199.80)]	= 0
PVF L Total (60 s)	11.85 (4.65)	2–15	Corrected score = raw score - [(1.3154)*(education − 13.52) + (−0.0345)*(education^2^ − 199.80)]	≤ 3.490
Perseveration L	0.76 (1.42)	0–3	-	≥ 4
Violation L	0.39 (0.952)	0–1	-	≥ 3
PVF total (60 s)	26.63 (9.12)	8–33	Corrected score = raw score - [(2.4758)*(education − 13.52) + (−0.06468)*(education^2^ − 199.80)]	≤ 10.135
PVF total perseverations	1.48 (2.59)	0–16	-	≥ 11
PVF total violations	0.61 (1.25)	0–7	-	≥ 5
SVF animals < 30 s	14.71 (3.69)	5–27	Corrected score = raw score - [(−0.0835)*(age − 60.07)]	≤ 7.328
SVF animals > 30 s	8.03 (3.48)	0–18	Corrected score = raw score - [(0.2039)*(education − 13.52)]	≤ 1.513
SVF animals Total (60 s)	22.74 (5.43)	12–34	Corrected score = raw score - [(−0.1073)*(age − 60.07) + (0.3155)*(education − 13.52)]	≤ 12.941
Perseveration animals	0.61 (1.18)	0–11	Corrected score = raw score - [(0.1967)*(age-60.07) + (−0.0016)*(age^2^- 3756.36)]	≥ 2.805
Violation animals	0.20 (0.62)	0–5	-	≥ 2
SVF vegetables < 30 s	10.04 (2.65)	3–16	Corrected score = raw score - [(2.2454)*(ln(100 - age) − 3.63) + (1.0097)*sex (F = 1, M = 0)]	≤ 4.543
SVF vegetables > 30 s	4.04 (2.25)	0–13	Corrected score = raw score - [(−0.0327)*(age − 60.07)]	≤ 0.356
SVF vegetables Total (60 s)	14.08 (3.64)	6–24	Corrected score = raw score - [(−0.0925)*(age − 60.07)]	≤ 7.566
Perseveration vegetables	0.62 (0.92)	0–4	-	≥ 3
Violation vegetables	1.01 (1.53)	0–7	Corrected score = raw score - [(0.0311)*(age − 60.07)]	≥ 4.597
SVF total (60 s)	36.82 (7.99)	19–57	Corrected score = raw score - [(−0.1974)*(age − 60.07) + (0.3558)*(education − 13.52)]	≤ 22.101
SVF total perseverations	1.22 (1.66)	0–13	Corrected score = raw score - [(0.2368)*(age − 60.07) + (−0.0019)*(age^2^ − 3756.36)]	≥ 5.274
SVF total violations	1.22 (1.66)	0–8	Corrected score = raw score − 0.03132*(age-60.07)	≥ 6.094
Benson Figure Copy	15.10 (1.9)	9–17	Corrected score = raw score - [(− 0.0538)*(age − 60.07) + (0.1066)*(education − 13.52)]	≤ 10.782
Benson Figure Recall	12.50 (2.8)	4–17	Corrected score = raw score - [(−1.0755)*sex(F = 1, M = 0) + (−0.0776)*(age − 60.07) + (0.1242)*(education − 13.52)]	≤ 6.969
Digit Span Forward: Score	7.04 (2.06)	2–13	Corrected score = raw score - [(0.4924)*(education − 13.52) + (0.0147)*(education^2^ − 199.80) −0.6536*sex (female = 1, Male = 0)	≤ 3.400
Digit Span Forward: Length	6.04 (1.16)	3–9	Corrected score = raw score - [(−0.0217)*(age − 60.07)]	≤ 3.868
Digit Span Backward: Score	6.61 (1.77)	3–11	Corrected score = raw score - [(−0.0258)*(age − 60.07) + (0.4413)*(education − 13.52) + (−0.0106)*(education^2^ − 199.80)]	≤ 3.340
Digit Span Backward: Length	4.78 (1.02)	3–7	Corrected score = raw score - [(−0.0128)*(age − 60.07) + (0.2956)*(education − 13.52) + (−0.0080)*(education^2^ − 199.80)]	≤ 2.978
TMT A (s)	39.99 (19.69)	14–166	Corrected score = raw score - [(−25.7849)*(ln(100 - age) − 3.63) + (−4.6087)*(education − 13.52) + (0.1364)*(education^2^ − 199.80)]	≥ 77.271
TMT B (s)	101.40 (49.12)	34–342	Corrected score = raw score - [(−53.5372)*(ln(100 - age) − 3.63) + (−51.1059)*(education − 13.52) + (2.9327)*(education^2^ − 199.80) + (−0.0533)*(education^3^ − 3182.83)]	≥ 174.034
TMT B-A (s)	61.41 (37.15)	6-235	Corrected score = raw score - [(0.8128)*(age − 60.07) + (−40.2412)*(education − 13.52) + (2.3557)*(education^2^ − 199.80) + (−0.0439)*(education^3^ − 3182.83)]	≥ 137.330

As shown, most of the tasks were influenced by age and education. Sex affected only the subscores of Semantic Verbal Fluency (Vegetables in the first 30 s) and Digit Span Forward test, with females performing better in the former and worse than males in the latter, respectively. It was not possible to carry out regression models on the Five Words test immediate and delayed total recall, violations, and perseverations in Phonological Verbal Ffluency (letter L, letter F, and total), violations (animals), and perseverations (vegetables and total) in the Semantic Verbal Fluency, due to reduced variability in these measures. Accordingly, the indicated cut-offs are derived directly from the raw scores.

Correction grids and Equivalent Scores are reported in Supplementary Table S1.

### Modality and order of administration effects

Supplementary Table 2 displays the results of the ANOVAs conducted to assess the effect of MODALITY (FACE-TO-FACE vs. REMOTE) and ORDER (FIRST vs. SECOND) on the performances of individuals on each test of the tele-I-UDSNB.

After correction for multiple comparisons, a significant effect of MODALITY was observed only on the score on Semantic Verbal Fluency – Animals (last 30 s) (p(FDR) = 0.005), with a higher score in the face-to-face modality. The effect size was fair (η^2^ = 0.153). No significant effect of ORDER was observed.

A significant effect of the interaction MODALITY x ORDER was observed on the number of violations on the Semantic Verbal Fluency test for the Animals category (*p* = 0.005), with a large effect size (η^2^ = 0.362). We have to note that data were moderately skewed in this sub-score, and no violations were reported for the individuals performing the test in remote modality as first administration (See also Supplementary Materials, Table S2).

## Discussion

The need for harmonized neuropsychological test batteries has emerged in recent years as a consequence of the increasing request for a reliable diagnosis for subjects in the prodromal phase of dementia, in particular of prodromal AD [4]. Furthermore, the recent pandemic made clear that remotely administered neuropsychological tools are crucial to grant access to diagnosis and follow-up in conditions in which mobility is reduced. Several neuropsychological tests that could be administered remotely have been developed (mainly through the adaptation of traditional tests) in the last decades [7]. In the case of the Italian-speaking population, in most cases the studies on remote assessment were devoted to the assessment of global cognition, using tests such as the telephone-based Mini-Mental State Examination (Itel-MMSE) [16, 17] or ad-hoc questionnaires [18]. More recently, tests assessing specific domains were adapted to remote administration [19, 20]. To our knowledge, the tele-I-UDSNB is the first comprehensive tele-neuropsychological battery to be standardized for the Italian population.

The results of the comparison between face-to-face and remote administration for the effect of demographic variables show that age consistently influenced scores obtained on most of the tests of the tele-I-UDSNB, in agreement with the results obtained in the face-to-face administration of I-UDSNB [5]. This was the case for verbal and visual episodic memory [21, 22]; memory tests with semantic cueing [23]; digit span [24]; Trail Making test [25]; copy of a complex figure [21]; naming [26, 27], and Semantic Verbal Fluency [28]. Education had a more limited effect, notably not observed in the Five Words test and in some of the sub-scores of the Semantic Verbal Fluency tests, in line with other test assessing single domains [23, 28]. The partial difference with the face-to-face administration of I-UDSNB may be due to limited statistical power, consequent to the reduced sample size and, in the case of the Five-word test, to the reduced number of items. The effect of sex was instead reported only in the Digit Span Forward test, with males outperforming women, and in the Vegetables Category Fluency task during the initial 30 s, with females outperforming men. These results are consistent with what we found in the in-person version of the I-UDSNB [5], and also with previous studies in Italian cohorts [29, 30].

Despite an overlap of the effect of demographic variables between face-to-face and remote administration on the performance of individuals, norms obtained on remote assessment show some significant differences. This finding is in line with the conclusions of a recent review of the literature [31], showing that the agreement between the two modalities of administration is good, but rarely perfect [12]. This line of evidence supports the need for specific norms and thresholds for remote testing [31].

The effect of the modality of administration (remote vs. face-to-face) was limited to a partial score of the Semantic Verbal Fluency task. In particular, the observed modality effect consisted of a reduced item generation in the last 30 s of the Animal fluency task during remote administration. No differences were found in total 60-seconds score or overall Semantic Verbal Fluency score, consistent with prior literature showing no significant impact of administration modality [7].

Finally, the repetition of both face-to-face and remote assessments was essential to examine the impact of administration modality. As no order effect was observed, a practice effect can be ruled out, supporting the reliability of the remote administration of the I-UDSNB. Parallel test could be introduced to further minimize practice effect, as recommended in previous research [32].

### Limitations

One limitation of the study is limited sample size. In particular, older participants were underrepresented, especially those with high levels of education — a pattern consistent with previous normative studies that have reported challenges in sampling this population, as also recently emphasized by Boccardi et al. [4]. Thus, we have to note the presence of constraints in the application of the findings to some demographic groups, such as older adults, but also individuals with limited access to computers or technology. This may impact the generalizability of the results and should be considered when interpreting the study implications. Finally, we have also to mention the presence of potential bias due to the imbalance in the administration order of tests.

## Conclusions

In conclusion, the tele-I-UDSNB may represent a reliable tool for the remote cognitive assessment of subjects with cognitive impairment. Validation studies assessing the diagnostic accuracy of the tele-I-UDSNB in detecting cognitive impairment will represent the further step needed to implement the use of this battery in clinical practice. Furthermore, within the Italian context, forthcoming initiatives may be directed toward the development of tailored modules for other forms of dementia, including dementia with Lewy bodies and frontotemporal lobar degeneration.

## Supplementary Information

Below is the link to the electronic supplementary material.

Supplementary Material 1

## Data Availability

The datasets used and/or analyzed during the current study are available from the corresponding author after a reasonable request.

## Abbreviations

AD: Alzheimer’s disease
ES: equivalent scores
F: female
M: male
PVF: Phonological Verbal Fluency
RIN: Italian Network of Neuroscience and Neuro-rehabilitation
s: seconds
SD: standard deviation
SVF: Semantic Verbal Fluency
TMT: Trail Making Test
UDSNB: Uniform Data Set – Neuropsychological Battery
