# Supplementary material for: The remote administration of the uniform data set neuropsychological test battery (I-UDSNB) Italian version: normative data
Source: Neurol Sci. 2025 Sep 20;46(11):5771–8. doi: 10.1007/s10072-025-08458-3 (PMC12537587; doi:10.1007/s10072-025-08458-3)
Supplement: Supplementary file 1 — Supplementary Material 1 [file 10072_2025_8458_MOESM1_ESM.docx]

**Supplementary materials**

**Table 1.** Correction grids. OTL: Outer Tolerance limit. ITL: inner tolerance limit.

| test | correction grid | | | |  | Equivalent Scores | | | | |
| --- | --- | --- | --- | --- | --- | --- | --- | --- | --- | --- |
| Craft Story |  |  |  |  |  |  | |  | | |
| Immediate verbatim score | age/education | ≤8 | 9-13 | ≥14 |  | 0 (OTL) | | ≤ 7.336 | | |
|  | 40-49 | 1.069 | -0.243 | -2.891 |  | ITL | | ≤ 9.593 | | |
|  | 50-59 | 2.030 | 0.717 | -1.931 |  | 1 | | ≤ 10.714 | | |
|  | 60-69 | 2.990 | 1.677 | -0.971 |  | 2 | | ≤ 13.262 | | |
|  | 70-79 | 3.950 | 2.637 | -0.011 |  | 3 | | ≤ 16.026 | | |
|  | 80-89 | 4.910 | 3.598 | 0.950 |  | 4 | | ≥16.027 | | |
| Recall verbatim score | age/education | ≤8 | 9-13 | ≥14 |  | 0 (OTL) | | ≤ 4.207 | | |
|  | 40-49 | 0.417 | -0.813 | -3.294 |  | ITL | | ≤ 7.116 | | |
|  | 50-59 | 1.705 | 0.475 | -2.006 |  | 1 | | ≤ 7.228 | | |
|  | 60-69 | 2.993 | 1.763 | -0.718 |  | 2 | | ≤ 10.090 | | |
|  | 70-79 | 4.281 | 3.051 | 0.569 |  | 3 | | ≤ 13.978 | | |
|  | 80-89 | 5.569 | 4.339 | 1.857 |  | 4 | | ≥13.979 | | |
| Five Words Test |  |  |  |  |  |  | |  | | |
| Immediate free recall | age |  |  |  |  | 0 (OTL) | | ≤ 2.963 | | |
|  | 40-49 | -0.183 |  |  |  | ITL | | ≤ 3.767 | | |
|  | 50-59 | -0.062 |  |  |  | 1 | | ≤ 3.768 | | |
|  | 60-69 | 0.060 |  |  |  | 2 | | ≤ 4.084 | | |
|  | 70-79 | 0.182 |  |  |  | 3 | | ≤ 4.853 | | |
|  | 80-89 | 0.303 |  |  |  | 4 | | ≥ 4.854 | | |
| Immediate cued recall | age |  |  |  |  |  | |  | | |
|  | 40-49 | 0.119 |  |  |  | 0 (OTL) | | ≥ 1.874 | | |
|  | 50-59 | 0.040 |  |  |  | ITL | | ≥ 1.135 | | |
|  | 60-69 | -0.039 |  |  |  |  | |  | | |
|  | 70-79 | -0.118 |  |  |  |  | |  | | |
|  | 80-89 | -0.197 |  |  |  |  | |  | | |
| Immediate total recall |  |  |  |  |  | 0 (OTL | | ≤ 4 | | |
|  |  |  |  |  |  | ITL | | ≤ 5 | | |
| Immediate total-weighted | age |  |  |  |  |  | |  | | |
|  | 40-49 | -0.247 |  |  |  | 0 (OTL) | | ≤ 7.114 | | |
|  | 50-59 | -0.083 |  |  |  | ITL | | ≤ 8.278 | | |
|  | 60-69 | 0.081 |  |  |  |  | |  | | |
|  | 70-79 | 0.245 |  |  |  |  | |  | | |
|  | 80-89 | 0.409 |  |  |  |  | |  | | |
| Delayed free recall | age |  |  |  |  |  | |  | | |
|  | 40-49 | -0.275 |  |  |  | 0 (OTL) | | ≤ 2.145 | | |
|  | 50-59 | -0.093 |  |  |  | ITL | | ≤ 3.109 | | |
|  | 60-69 | 0.090 |  |  | | |  | | | |
|  | 70-79 | 0.273 |  |  |  |  | | | |  |
|  | 80-89 | 0.455 |  |  |  |  | | | |  |
| Delayed cued recall | age |  |  |  |  |  | |  | | |
|  | 40-49 | 0.097 |  |  |  | 0 (OTL) | | ≥ 2.009 | | |
|  | 50-59 | 0.045 |  |  |  | ITL | | ≥ 1.101 | | |
|  | 60-69 | -0.019 |  |  |  |  | |  | | |
|  | 70-79 | -0.105 |  |  |  |  | |  | | |
|  | 80-89 | -0.236 |  |  |  |  | |  | | |
| Delayed total recall |  |  |  |  |  | 0 (OTL) | | ≤ 3 | | |
|  |  |  |  |  |  | ITL | | ≤ 4 | | |
| Delayed total-weighted | age |  |  |  |  | | |  | | |
|  | 40-49 | -0.441 |  |  | 0 (OTL) | | | ≤ 6.027 | | |
|  | 50-59 | -0.148 |  |  |  | ITL | | ≤ 7.232 | | |
|  | 60-69 | 0.144 |  |  |  | | |  | | |
|  | 70-79 | 0.437 |  |  |  | | |  | | |
|  | 80-89 | 0.730 |  |  |  |  | |  | | |
| Total free recall | age |  |  |  |  | 0 (OTL) | | ≤ 6.049 | | |
|  | 40-49 | -0.403 |  |  |  | ITL | | ≤ 6.860 | | |
|  | 50-59 | -0.189 |  |  |  | 1 | | ≤ 7.110 | | |
|  | 60-69 | 0.079 |  |  |  | 2 | | ≤ 8.504 | | |
|  | 70-79 | 0.439 |  |  |  | 3 | | ≤ 9.079 | | |
|  | 80-89 | 0.984 |  |  |  | 4 | | ≥ 9.080 | | |
| Total cued recall | age |  |  |  |  | 0 (OTL) | | ≥ 3.153 | | |
|  | 40-49 | 0.229 |  |  |  | ITL | | ≥ 2.229 | | |
|  | 50-59 | 0.077 |  |  |  |  | |  | | |
|  | 60-69 | -0.075 |  |  |  |  | |  | | |
|  | 70-79 | -0.227 |  |  |  |  | |  | | |
|  | 80-89 | -0.378 |  |  |  |  | |  | | |
| Total recall | age |  |  |  |  |  | |  | | |
|  | 40-49 | -0.230 |  |  |  | 0 (OTL) | | ≤ 8.090 | | |
|  | 50-59 | -0.077 |  |  |  | ITL | | ≤ 8.756 | | |
|  | 60-69 | 0.075 |  |  |  |  | |  | | |
|  | 70-79 | 0.228 |  |  |  |  | |  | | |
|  | 80-89 | 0.380 |  |  |  |  | |  | | |
| Total-weighted recall | age |  |  |  |  | 0 (OTL) | | ≤ 14.418 | | |
|  | 40-49 | -0.602 |  |  |  | ITL | | ≤ 15.791 | | |
|  | 50-59 | -0.282 |  |  |  | 1 | | ≤ 15.964 | | |
|  | 60-69 | 0.118 |  |  |  | 2 | | ≤ 17.906 | | |
|  | 70-79 | 0.655 |  |  |  | 3 | | ≤ 19.118 | | |
|  | 80-89 | 1.469 |  |  |  | 4 | | ≥ 19.119 | | |
| Picture Naming |  |  |  |  |  |  | |  | | |
| Correct without cue score | age |  |  |  |  | 0 (OTL) | | ≤ 28.225 | | |
|  | 40-49 | -0.571 |  |  |  | ITL | | ≤ 30.277 | | |
|  | 50-59 | -0.192 |  |  |  |  |  |  |  |  |
|  | 60-69 | 0.187 |  |  |  |  |  |  |  |  |
|  | 70-79 | 0.566 |  |  |  |  |  |  |  |  |
|  | 80-89 | 0.945 |  |  |  |  |  |  |  |  |
| Correct with cue score | age |  |  |  |  | 0 (OTL) | | ≥ 1.419 | | |
|  | 40-49 | 0.137 |  |  |  | ITL | | ≥ 0.492 | | |
|  | 50-59 | 0.046 |  |  |  |  | |  | | |
|  | 60-69 | -0.045 |  |  |  |  | |  | | |
|  | 70-79 | -0.136 |  |  |  |  | |  | | |
|  | 80-89 | -0.227 |  |  |  |  | |  | | |
| Correct total score | age |  |  |  |  | 0 (OTL) | | ≤ 28.665 | | |
|  | 40-49 | -0.420 |  |  |  | ITL | | ≤ 30.580 | | |
|  | 50-59 | -0.141 |  |  |  |  | |  | | |
|  | 60-69 | 0.137 |  |  |  |  | |  | | |
|  | 70-79 | 0.416 |  |  |  |  | |  | | |
|  | 80-89 | 0.695 |  |  |  |  | |  | | |
| Semantic Fluency |  |  |  |  |  |  | |  | | |
| Animals correct score (< 30 s) | age |  |  |  | 0 (OTL) | | | ≤ 7.328 |  | |
|  | 40-49 | -1.259 |  |  |  | ITL | | ≤ 10.491 | | |
|  | 50-59 | -0.423 |  |  |  | 1 | | ≤ 10.579 | | |
|  | 60-69 | 0.412 |  |  |  | 2 | | ≤ 12.414 | | |
|  | 70-79 | 1.247 |  |  |  | 3 | | ≤ 14.412 | | |
|  | 80-89 | 2.082 |  |  |  | 4 | | ≥ 14.413 | | |
| Animals correct score (> 30 s) | education | ≤8 | 9-13 | >13 |  | 0 (OTL) | | ≤ 1.513 | | |
|  |  | 1.533 | 0.513 | -0.303 |  | ITL | | ≤ 3.290 | | |
|  |  |  |  |  |  | 1 | | ≤ 4.105 | | |
|  |  |  |  |  |  | 2 | | ≤ 5.493 | | |
|  |  |  |  |  |  | 3 | | ≤ 7.290 | | |
|  |  |  |  |  |  | 4 | | ≥ 7.291 | | |
| Animals total correct score (60 s) | age/education | ≤8 | 9-13 | >13 |  | 0 (OTL) | | ≤ 12.941 | | |
|  | 40-49 | 0.754 | -0.824 | -2.086 |  | ITL | | ≤ 15.324 | | |
|  | 50-59 | 1.827 | 0.249 | -1.013 |  | 1 | | ≤ 15.652 | | |
|  | 60-69 | 2.900 | 1.322 | 0.060 |  | 2 | | ≤ 19.116 | | |
|  | 70-79 | 3.972 | 2.395 | 1.132 |  | 3 | | ≤ 22.449 | | |
|  | 80-89 | 5.045 | 3.467 | 2.205 |  | 4 | | ≥ 20.450 | | |
| Animals violations |  |  |  |  |  | 0 (OTL) | | ≥ 2 | | |
|  |  |  |  |  |  | ITL | | ≥ 1 | | |
| Animals perseveration | age |  |  |  |  | 0 (OTL) | | ≥ 2.805 | | |
|  | 40-49 | 0.204 |  |  |  | ITL | | ≥ 1.851 | | |
|  | 50-59 | -0.169 |  |  |  | 1 | | ≥ 1.812 | | |
|  | 60-69 | -0.223 |  |  |  | 2 | | ≥ 0.898 | | |
|  | 70-79 | 0.042 |  |  |  | 3 | | 0 | | |
|  | 80-89 | 0.623 |  |  |  | 4 | | 0 | | |
| Vegetables correct score (< 30 s) | age/ sex | F | M |  |  | 0 (OTL) | | ≤ 4.543 | | |
|  | 40-49 | -1.858 | -0.848 |  |  | ITL | | ≤ 5.925 | | |
|  | 50-59 | -1.407 | -0.398 |  |  | 1 | | ≤ 6.100 | | |
|  | 60-69 | -0.843 | 0.167 |  |  | 2 | | ≤ 7.857 | | |
|  | 70-79 | -0.087 | 0.922 |  |  | 3 | | ≤ 9.157 | | |
|  | 80-89 | 1.060 | 2.069 |  |  | 4 | | ≥ 9.158 | | |
| Vegetables correct score (> 30 s) | age |  |  |  |  | 0 (OTL) | | ≤ 0.356 | | |
|  | 40-49 | -0.533 |  |  |  | ITL | | ≤ 1.194 | | |
|  | 50-59 | -0.186 |  |  |  | 1 | | ≤ 1.402 | | |
|  | 60-69 | 0.141 |  |  |  | 2 | | ≤ 2.533 | | |
|  | 70-79 | 0.467 |  |  |  | 3 | | ≤ 3.519 | | |
|  | 80-89 | 0.794 |  |  |  | 4 | | ≥ 3.520 | | |
| Vegetables total correct score (60 s) | age |  |  |  |  | 0 (OTL) | | ≤ 7.566 | | |
|  | 40-49 | -1.394 |  |  |  | ITL | | ≤ 9.606 | | |
|  | 50-59 | -0.469 |  |  |  | 1 | | ≤9.716 | | |
|  | 60-69 | 0.456 |  |  |  | 2 | | ≤ 11.676 | | |
|  | 70-79 | 1.381 |  |  |  | 3 | | ≤ 13.844 | | |
|  | 80-89 | 2.306 |  |  |  | 4 | | ≥ 13.845 | | |
| Vegetables perseverations |  |  |  |  |  | 0 (OTL) | | ≥ 3 | | |
|  |  |  |  |  |  | ITL | | ≥ 2 | | |
| Vegetables violations | age |  |  |  |  | 0 (OTL) | | ≥ 4.598 | | |
|  | 40-49 | 0.469 |  |  |  | ITL | | ≥ 3.505 | | |
|  | 50-59 | 0.158 |  |  |  | 1 | | ≥ 3.251 | | |
|  | 60-69 | -0.153 |  |  |  | 2 | | ≥ 1.469 | | |
|  | 70-79 | -0.465 |  |  |  | 3 | | 0 | | |
|  | 80-89 | -0.776 |  |  |  | 4 | | 0 | | |
| Total correct score (60 s) | age/education | ≤8 | 9-13 | >13 |  | 0 (OTL) | | ≤ 22.101 | | |
|  | 40-49 | -0.302 | -2.081 | -3.504 |  | ITL | | ≤ 25.984 | | |
|  | 50-59 | 1.672 | -0.107 | -1.530 |  | 1 | | ≤ 26.737 | | |
|  | 60-69 | 3.646 | 1.867 | 0.444 |  | 2 | | ≤ 32.010 | | |
|  | 70-79 | 5.620 | 3.841 | 2.418 |  | 3 | | ≤ 36.982 | | |
|  | 80-89 | 7.594 | 5.815 | 4.392 |  | 4 | | ≥ 36.983 | | |
| Total violations | age |  |  |  |  | 0 (OTL) | | ≥ 6.094 | | |
|  | 40-49 | 0.209 |  |  |  | ITL | | ≥ 3.215 | | |
|  | 50-59 | -0.218 |  |  |  | 1 | | ≥ 3.752 | | |
|  | 60-69 | -0.258 |  |  |  | 2 | | ≥ 1.908 | | |
|  | 70-79 | 0.090 |  |  |  | 3 | | ≥ 0.658 | | |
|  | 80-89 | 0.826 |  |  |  | 4 | | ≤ 0.657 | | |
| Total perseverations | age |  |  |  |  | 0 (OTL) | | ≥ 5.274 | | |
|  | 40-49 | 0.209 |  |  |  | ITL | | ≥ 3.215 | | |
|  | 50-59 | -0.218 |  |  |  | 1 | | ≥ 3.093 | | |
|  | 60-69 | -0.258 |  |  |  | 2 | | ≥ 1.782 | | |
|  | 70-79 | 0.090 |  |  |  | 3 | | ≥ 0.782 | | |
|  | 80-89 | 0.826 |  |  |  | 4 | | ≤ 0.781 | | |
| Phonemic Fluency |  |  |  |  |  |  | |  | | |
| Letter F correct score (< 30 s) | education | ≤8 | 9-13 | >13 |  | 0 (OTL) | | ≤ 3.136 | | |
|  |  | 1.548 | 0.518 | -0.306 |  | ITL | | ≤ 4.841 | | |
|  |  |  |  |  |  | 1 | | ≤ 5.076 | | |
|  |  |  |  |  |  | 2 | | ≤ 7.106 | | |
|  |  |  |  |  |  | 3 | | ≤ 9.076 | | |
|  |  |  |  |  |  | 4 | | ≥ 9.077 | | |
| Letter F correct score (> 30 s) | education | ≤8 | 9-13 | >13 |  | 0 (OTL) | | ≤ 0.370 | | |
|  |  | 1.056 | 0.354 | -0.209 |  | ITL | | ≤ 2.073 | | |
|  |  |  |  |  |  | 1 | | ≤ 2.354 | | |
|  |  |  |  |  |  | 2 | | ≤ 4.073 | | |
|  |  |  |  |  |  | 3 | | ≤ 5.510 | | |
|  |  |  |  |  |  | 4 | | ≥ 5.511 | | |
| Letter F total correct score (60 s) | education | ≤8 | 9-13 | >13 |  | 0 (OTL) | | ≤ 5.539 | | |
|  |  | 3.786 | 0.545 | -0.963 |  | ITL | | ≤ 8.647 | | |
|  |  |  |  |  |  | 1 | | ≤ 8.671 | | |
|  |  |  |  |  |  | 2 | | ≤ 11.545 | | |
|  |  |  |  |  |  | 3 | | ≤ 14.539 | | |
|  |  |  |  |  |  | 4 | | ≥ 14.540 | | |
| Letter F violation |  |  |  |  |  | 0 (OTL) | | ≥ 2 | | |
|  |  |  |  |  |  | ITL | | ≥ 1 | | |
| Letter F perseveration |  |  |  |  |  | 0 (OTL) | | ≥ 5 | | |
|  |  |  |  |  |  | ITL | | ≥ 3 | | |
| Letter L correct score (< 30 s) | education | ≤8 | 9-13 | >13 |  | 0 (OTL) | | ≤ 2.245 | | |
|  |  | 2.402 | 0.349 | -0.610 |  | ITL | | ≤ 3.796 | | |
|  |  |  |  |  |  | 1 | | ≤ 4.052 | | |
|  |  |  |  |  |  | 2 | | ≤ 5.793 | | |
|  |  |  |  |  |  | 3 | | ≤ 7.139 | | |
|  |  |  |  |  |  | 4 | | ≥ 7.140 | | |
| Letter L correct score (> 30 s) | education | ≤8 | 9-13 | >13 |  | 0 (OTL) | | 0 | | |
|  |  | 1.841 | 0.246 | -0.474 |  | ITL | | ≤ 1.358 | | |
|  |  |  |  |  |  | 1 | | ≤ 1.824 | | |
|  |  |  |  |  |  | 2 | | ≤ 2.824 | | |
|  |  |  |  |  |  | 3 | | ≤ 4.111 | | |
|  |  |  |  |  |  | 4 | | ≥ 4.112 | | |
| Letter L total correct score (60 s) | education | ≤8 | 9-13 | >13 |  | 0 (OTL) | | ≤ 3.49 | | |
|  |  | 4.244 | 0.595 | -1.084 |  | ITL | | ≤ 6.599 | | |
|  |  |  |  |  |  | 1 | | ≤ 6.621 | | |
|  |  |  |  |  |  | 2 | | ≤ 9.238 | | |
|  |  |  |  |  |  | 3 | | ≤ 11.499 | | |
|  |  |  |  |  |  | 4 | | ≥ 11.500 | | |
| Letter L perseveration |  |  |  |  |  | 0 (OTL) | | ≥ 4 | | |
|  |  |  |  |  |  | ITL | | ≥ 3 | | |
| Letter L violation |  |  |  |  |  | 0 (OTL) | | ≥ 3 | | |
|  |  |  |  |  |  | ITL | | ≥ 2 | | |
| Total correct score (60 s) | education | ≤8 | 9-13 | >13 |  | 0 (OTL) | | ≤ 10.135 | | |
|  |  | 8.030 | 1.140 | -2.046 |  | ITL | | ≤ 15.883 | | |
|  |  |  |  |  |  | 1 | | ≤16.140 | | |
|  |  |  |  |  |  | 2 | | ≤ 20.150 | | |
|  |  |  |  |  |  | 3 | | ≤ 26.140 | | |
|  |  |  |  |  |  | 4 | | ≥ 26.141 | | |
| Total perseverations |  |  |  |  |  | 0 (OTL) | | ≥ 11 | | |
|  |  |  |  |  |  | ITL | | ≥ 4 | | |
| Total violations |  |  |  |  |  | 0 (OTL) | | ≥ 5 | | |
|  |  |  |  |  |  | ITL | | ≥ 2 | | |
| Benson Figure |  |  |  |  |  |  | |  | | |
| Copy | age/education | ≤8 | 9-13 | >13 |  | 0 (OTL) | | ≤ 10.782 | | |
|  | 40-49 | -0.096 | -0.543 | -1.215 |  | ITL | | ≤ 12.558 | | |
|  | 50-59 | 0.443 | -0.005 | -0.676 |  | 1 | | ≤ 12.584 | | |
|  | 60-69 | 0.981 | 0.534 | -0.138 |  | 2 | | ≤ 13.890 | | |
|  | 70-79 | 1.520 | 1.072 | 0.400 |  | 3 | | ≤ 15.325 | | |
|  | 80-89 | 2.058 | 1.610 | 0.939 |  | 4 | | ≥ 15.326 | | |
| Recall | Female/ age/education | ≤8 | 9-13 | >13 |  |  | |  | | |
|  | 40-49 | 0.111 | -0.411 | -1.194 |  |  | |  | | |
|  | 50-59 | 0.886 | 0.364 | -0.418 |  |  | |  | | |
|  | 60-69 | 1.662 | 1.140 | 0.357 |  | 0 (OTL) | | ≤ 6.969 | | |
|  | 70-79 | 2.437 | 1.915 | 1.133 |  | ITL | | ≤ 8.505 | | |
|  | 80-89 | 3.213 | 2.691 | 1.908 |  | 1 | | ≤ 8.611 | | |
|  | Male/ age/education | ≤8 | 9-13 | ≥14 |  | 2 | | ≤ 11.202 | | |
|  | 40-49 | -2.040 | -2.562 | -3.345 |  | 3 | | ≤ 12.719 | | |
|  | 50-59 | -1.265 | -1.787 | -2.569 |  | 4 | | ≥ 12.720 | | |
|  | 60-69 | -0.489 | -1.011 | -1.794 |  |  | |  | | |
|  | 70-79 | 0.286 | -0.236 | -1.018 |  |  | |  | | |
|  | 80-89 | 1.062 | 0.540 | -0.243 |  |  | |  | | |
| Digit Span Forward |  |  |  |  |  |  | |  | | |
| Number of correct trials | Female, education | ≤8 | 9-13 | >13 |  |  | |  | | |
|  |  | 1.379 | -0.627 | -3.636 |  |  | |  | | |
|  |  |  |  |  |  |  | |  | | |
|  |  |  |  |  |  | 0 (OTL) | | ≤ 3.400 | | |
|  |  |  |  |  |  | ITL | | ≤ 4.450 | | |
|  |  |  |  |  |  | 1 | | ≤ 5.191 | | |
|  | Male, education | ≤8 | 9-13 | >13 |  | 2 | | ≤ 7.400 | | |
|  |  | 0.072 | -1.934 | -4.944 |  | 3 | | ≤ 11.191 | | |
|  |  |  |  |  |  | 4 | | ≥ 11.192 | | |
|  |  |  |  |  |  |  | |  | | |
|  |  |  |  |  |  |  | |  | | |
|  |  |  |  |  |  |  | |  | | |
| Span length | age |  |  |  |  | 0 (OTL) | | ≤ 3.868 | | |
|  | 40-49 | -0.327 |  |  |  | ITL | | ≤ 4.586 | | |
|  | 50-59 | -0.110 |  |  |  | 1 | | ≤ 4.717 | | |
|  | 60-69 | 0.107 |  |  |  | 2 | | ≤ 5.652 | | |
|  | 70-79 | 0.324 |  |  |  | 3 | | ≤ 6.890 | | |
|  | 80-89 | 0.540 |  |  |  | 4 | | ≥ 6.891 | | |
| Digit Span Backward |  |  |  |  |  |  | |  | | |
| Number of correct trials | age/education | ≤8 | 9-13 | >13 |  | 0 (OTL) | | ≤ 3.340 | | |
|  | 40-49 | 1.132 | 0.069 | -0.827 |  | ITL | | ≤ 3.927 | | |
|  | 50-59 | 1.391 | 0.327 | -0.568 |  | 1 | | ≤ 4.236 | | |
|  | 60-69 | 1.649 | 0.586 | -0.310 |  | 2 | | ≤ 5.920 | | |
|  | 70-79 | 1.907 | 0.844 | -0.052 |  | 3 | | ≤ 6.956 | | |
|  | 80-89 | 2.166 | 1.102 | 0.207 |  | 4 | | ≥ 6.957 | | |
| Span length | age/education | ≤8 | 9-13 | >13 |  | 0 (OTL) | | ≤ 2.978 | | |
|  | 40-49 | 0.696 | 0.055 | -0.375 |  | ITL | | ≤ 3.479 | | |
|  | 50-59 | 0.824 | 0.183 | -0.247 |  | 1 | | ≤ 3.849 | | |
|  | 60-69 | 0.952 | 0.311 | -0.119 |  | 2 | | ≤ 4.432 | | |
|  | 70-79 | 1.081 | 0.440 | 0.010 |  | 3 | | ≤ 4.990 | | |
|  | 80-89 | 1.209 | 0.568 | 0.138 |  | 4 | | ≥ 4.991 | | |
| Trial Making Test |  |  |  |  |  |  | |  | | |
| Part A (seconds) | age/education | ≤8 | 9-13 | >13 |  | 0 (OTL) | | ≥ 77.271 | | |
|  | 40-49 | -4.087 | 5.073 | 9.793 |  | ITL | | ≥ 62.132 | | |
|  | 50-59 | -9.261 | -0.101 | 4.619 |  | 1 | | ≥ 54.592 | | |
|  | 60-69 | -15.742 | -6.581 | -1.862 |  | 2 | | ≥ 41.740 | | |
|  | 70-79 | -24.418 | -15.257 | -10.538 |  | 3 | | ≥ 33.425 | | |
|  | 80-89 | -37.589 | -28.429 | -23.709 |  | 4 | | ≤ 33.424 | | |
| Part B (seconds) | age/education | ≤8 | 9-13 | >13 |  | 0 (OTL) | | ≥ 174.034 | | |
|  | 40-49 | -62.740 | -13.118 | -8.830 |  | ITL | | ≥ 144.644 | | |
|  | 50-59 | -73.484 | -23.861 | -19.574 |  | 1 | | ≥ 128.150 | | |
|  | 60-69 | -86.938 | -37.316 | -33.028 |  | 2 | | ≥ 104.967 | | |
|  | 70-79 | -104.952 | -55.330 | -51.042 |  | 3 | | ≥ 85.776 | | |
|  | 80-89 | -132.300 | -82.678 | -78.390 |  | 4 | | ≤ 85.775 | | |
| Part B-A (seconds) | age/education | ≤8 | 9-13 | >13 |  | 0 (OTL) | | ≥ 137.330 | | |
|  | 40-49 | -31.135 | 6.370 | 8.672 |  | ITL | | ≥ 100.473 | | |
|  | 50-59 | -77.104 | -39.599 | -37.297 |  | 1 | | ≥ 83.710 | | |
|  | 60-69 | -96.219 | -58.714 | -56.412 |  | 2 | | ≥ 67.033 | | |
|  | 70-79 | -104.348 | -66.843 | -64.540 |  | 3 | | ≥ 47.580 | | |
|  | 80-89 | -112.476 | -74.971 | -72.669 |  | 4 | | ≤ 47.579 | | |

**Table 2.** ANOVAs on scores obtained in respect of order and modality of administration of the Tele-I-UDSNB. PVF: Phonological Verbal Fluency; s: seconds; SVF: Semantic Verbal Fluency; TMT: Trail Making Test.

|  | FACE-TO-FACE | | | | REMOTE | | | |  |  |  |  |  |  |  |  |  |  |  |  |
| --- | --- | --- | --- | --- | --- | --- | --- | --- | --- | --- | --- | --- | --- | --- | --- | --- | --- | --- | --- | --- |
|  | ORDER | | | | ORDER | | | |  |  |  |  |  |  |  |  |  |  |  |  |
|  | FIRST | | SECOND | | FIRST | | SECOND | | MODALITY | | | | ORDER | | | | MODALITY X ORDER | | | |
|  | Mean | Std. Deviation | Mean | Std. Deviation | Mean | Std. Deviation | Mean | Std. Deviation | F | p | p (FDR) | partial eta2 | F | p | p (FDR) | partial eta2 | F | p | p (FDR) | partial eta2 |
| **Benson Figure Copy** | 14.35 | 1.309 | 15.60 | .598 | 14.85 | 1.348 | 14.50 | 1.821 | 1.020 | .316 | 0.868 | .013 | 2.294 | .134 | 0.708 | .030 | 5.470 | **.022** | 0.233 | .068 |
| **Benson Figure Recall** | 11.10 | 2.808 | 12.85 | 2.943 | 12.35 | 2.834 | 11.35 | 3.167 | .036 | .851 | 0.945 | .000 | 0.321 | .573 | 0.841 | .004 | .300 | .585 | 0.872 | .004 |
| **5 words immediate free recall** | 4.50 | .827 | 4.65 | .489 | 4.60 | .598 | 4.65 | .587 | .126 | .724 | 0.945 | .002 | 0.503 | .480 | 0.841 | .007 | 2.149 | .147 | 0.708 | .028 |
| **5 words immediate cued recall** | .50 | .827 | .30 | .470 | .40 | .598 | .25 | .550 | .289 | .593 | 0.945 | .004 | 1.572 | .214 | 0.709 | .021 | 1.129 | .291 | 0.826 | .015 |
| **5 words immediate total recall** | 5.00 | 0.000 | 4.95 | .224 | 5.00 | 0.000 | 4.90 | .308 | .353 | .554 | 0.945 | .005 | 3.179 | .079 | 0.708 | .041 | 1.928 | .169 | 0.746 | .025 |
| **5 words immediate total-weighted** | 9.50 | .827 | 9.60 | .598 | 9.60 | .598 | 9.55 | .759 | .026 | .872 | 0.945 | .000 | 0.026 | .872 | 0.906 | .000 | 2.951 | .090 | 0.530 | .038 |
| **5 words Delayed free recall** | 3.95 | 1.099 | 4.65 | .587 | 4.60 | .503 | 4.40 | .821 | 1.298 | .258 | 0.868 | .017 | 2.028 | .159 | 0.708 | .026 | .124 | .726 | 0.890 | .002 |
| **5 words Delayed cued recall** | .85 | .988 | .30 | .571 | .35 | .489 | .45 | .686 | 1.218 | .273 | 0.868 | .016 | 2.014 | .160 | 0.708 | .026 | .131 | .719 | 0.890 | .002 |
| **5 words Delayed total recall** | 4.80 | .410 | 4.95 | .224 | 4.95 | .224 | 4.85 | .366 | .123 | .727 | 0.945 | .002 | 0.123 | .727 | 0.863 | .002 | .004 | .951 | 0.969 | .000 |
| **5 words Delayed total-weighted** | 8.75 | 1.333 | 9.60 | .681 | 9.55 | .605 | 9.25 | 1.070 | 1.085 | .301 | 0.868 | .014 | 1.621 | .207 | 0.709 | .021 | .094 | .760 | 0.895 | .001 |
| **5 words Total free recall** | 8.45 | 1.572 | 9.30 | .801 | 9.20 | .894 | 9.05 | 1.317 | .907 | .344 | 0.868 | .012 | 1.777 | .187 | 0.708 | .023 | 1.045 | .310 | 0.826 | .014 |
| **5 words Total cued recall** | 1.35 | 1.387 | .60 | .754 | .75 | .910 | .70 | 1.174 | 1.076 | .303 | 0.868 | .014 | 2.754 | .101 | 0.708 | .035 | .727 | .396 | 0.826 | .010 |
| **5 words Total recall** | 9.80 | .410 | 9.90 | .308 | 9.95 | .224 | 9.75 | .550 | 0.000 | 1.000 | 1.000 | 0.000 | 0.328 | .569 | 0.841 | .004 | .517 | .474 | 0.837 | .007 |
| **5 words Total-weighted recall** | 18.25 | 1.832 | 19.20 | .951 | 19.15 | .933 | 18.80 | 1.642 | .658 | .420 | 0.928 | .009 | 0.947 | .334 | 0.841 | .012 | 1.155 | .286 | 0.826 | .015 |
| **Craft story immediate verbatim** | 15.30 | 6.674 | 19.70 | 5.573 | 17.55 | 4.850 | 17.10 | 6.537 | .018 | .893 | 0.945 | .000 | 2.316 | .132 | 0.708 | .030 | 2.441 | .122 | 0.647 | .032 |
| **Craft story recall verbatim** | 13.65 | 5.941 | 18.65 | 6.055 | 15.55 | 5.671 | 14.70 | 7.042 | .603 | .440 | 0.933 | .008 | 2.471 | .120 | 0.708 | .032 | 4.612 | **.035** | 0.309 | .058 |
| **Digit Span Forward: Score** | 6.65 | 1.725 | 6.60 | 1.984 | 6.50 | 1.606 | 7.15 | 2.084 | .228 | .634 | 0.945 | .003 | 0.514 | .476 | 0.841 | .007 | .174 | .678 | 0.890 | .002 |
| **Digit Span Forward: lenght** | 5.80 | .834 | 5.90 | 1.373 | 5.75 | 1.020 | 6.10 | 1.210 | .088 | .768 | 0.945 | .001 | 0.792 | .376 | 0.841 | .010 | .707 | .403 | 0.826 | .009 |
| **Digit Span Backward: Score** | 5.95 | 1.701 | 6.20 | 1.281 | 5.95 | 1.317 | 6.70 | 1.625 | .554 | .459 | 0.936 | .007 | 2.215 | .141 | 0.708 | .029 | .113 | .737 | 0.890 | .002 |
| **Digit Span Backward: lenght** | 4.35 | .988 | 4.75 | .851 | 4.60 | .821 | 4.80 | 1.152 | .480 | .490 | 0.945 | .006 | 1.921 | .170 | 0.708 | .025 | .056 | .813 | 0.916 | .001 |
| **PVF F <30** | 10.25 | 2.751 | 10.40 | 2.798 | 7.90 | 2.882 | 9.30 | 3.585 | 6.950 | **.010** | 0.265 | .085 | 1.403 | .240 | 0.748 | .018 | 7.005 | **.010** | 0.133 | .085 |
| **PVF F >30** | 5.80 | 3.037 | 5.90 | 2.125 | 5.95 | 1.538 | 6.30 | 2.904 | .243 | .623 | 0.945 | .003 | 0.163 | .688 | 0.863 | .002 | .013 | .909 | 0.955 | .000 |
| **PVF F Total** | 16.05 | 4.501 | 16.30 | 4.305 | 13.85 | 3.329 | 15.60 | 5.670 | 2.096 | .152 | 0.868 | .027 | 0.997 | .321 | 0.841 | .013 | 3.214 | .077 | 0.510 | .041 |
| **Perseveration F** | .95 | .999 | .45 | .686 | .25 | .550 | .50 | 1.000 | 3.052 | .085 | 0.868 | .039 | 0.452 | .504 | 0.841 | .006 | .138 | .711 | 0.890 | .002 |
| **Violation F** | .65 | 1.137 | 0.00 | 0.000 | 0.00 | 0.000 | .50 | .889 | .213 | .645 | 0.945 | .003 | 0.213 | .645 | 0.863 | .003 | .963 | .330 | 0.826 | .013 |
| **PVF L <30** | 8.60 | 3.267 | 8.15 | 2.498 | 6.80 | 1.609 | 7.60 | 3.440 | 3.490 | .066 | 0.868 | .044 | 0.077 | .782 | 0.863 | .001 | .589 | .445 | 0.826 | .008 |
| **PVF L >30** | 5.10 | 2.594 | 4.10 | 1.861 | 3.70 | 2.342 | 5.65 | 2.621 | .020 | .888 | 0.945 | .000 | 0.795 | .376 | 0.841 | .010 | .046 | .830 | 0.916 | .001 |
| **PVF L Total** | 13.70 | 5.100 | 12.25 | 3.823 | 10.50 | 3.332 | 13.25 | 5.486 | 1.167 | .283 | 0.868 | .015 | 0.408 | .525 | 0.841 | .005 | .344 | .559 | 0.872 | .005 |
| **Perseveration L** | .65 | .988 | .35 | .587 | .45 | .887 | .60 | .940 | .016 | .898 | 0.945 | .000 | 0.148 | .701 | 0.863 | .002 | .037 | .848 | 0.917 | .000 |
| **Violation L** | .80 | 1.240 | 0.00 | 0.000 | 0.00 | 0.000 | .75 | 1.482 | .013 | .909 | 0.945 | .000 | 0.013 | .909 | 0.926 | .000 | .646 | .424 | 0.826 | .009 |
| **PVF total** | 29.75 | 8.644 | 28.55 | 7.265 | 24.35 | 5.950 | 28.85 | 10.559 | 1.889 | .173 | 0.868 | .025 | 0.791 | .377 | 0.841 | .010 | 1.664 | .201 | 0.819 | .022 |
| **PVF total perseverations** | 1.60 | 1.698 | .80 | 1.152 | .70 | 1.129 | 1.10 | 1.518 | .918 | .341 | 0.868 | .012 | 0.408 | .525 | 0.841 | .005 | .010 | .919 | 0.955 | .000 |
| **PVF total violations** | 1.45 | 2.089 | 0.00 | 0.000 | 0.00 | 0.000 | 1.25 | 2.023 | .093 | .761 | 0.945 | .001 | 0.093 | .761 | 0.863 | .001 | 1.042 | .311 | 0.826 | .014 |
| **SVF animals <30** | 13.80 | 2.984 | 13.65 | 3.760 | 14.50 | 4.674 | 15.10 | 2.614 | 1.765 | .188 | 0.868 | .023 | 0.077 | .782 | 0.863 | .001 | .060 | .806 | 0.916 | .001 |
| **SVF animals >30** | 9.05 | 3.620 | 9.60 | 2.927 | 6.80 | 2.802 | 6.55 | 3.426 | 13.572 | **.000** | **0.005** | .153 | 0.043 | .835 | 0.885 | .001 | .430 | .514 | 0.872 | .006 |
| **SVF animals Total** | 22.85 | 5.314 | 23.25 | 4.598 | 21.30 | 5.059 | 21.65 | 4.987 | 1.972 | .164 | 0.868 | .026 | 0.112 | .739 | 0.863 | .001 | .358 | .552 | 0.872 | .005 |
| **Perseveration animals** | .70 | .923 | .55 | .887 | .55 | .686 | .50 | .761 | .297 | .587 | 0.945 | .004 | 0.297 | .587 | 0.841 | .004 | .669 | .416 | 0.826 | .009 |
| **Violation animals** | .40 | 1.188 | .15 | .366 | 0.00 | 0.000 | .50 | 1.192 | .025 | .875 | 0.945 | .000 | 0.622 | .433 | 0.841 | .008 | 42.530 | **.000** | **0.005** | .362 |
| **SVF vegetables <30** | 10.30 | 3.686 | 9.15 | 2.925 | 9.40 | 2.542 | 9.80 | 2.546 | .035 | .852 | 0.945 | .000 | 0.317 | .575 | 0.841 | .004 | .571 | .452 | 0.826 | .008 |
| **SVF vegetables >30** | 4.20 | 3.088 | 4.30 | 2.055 | 3.45 | 1.731 | 4.00 | 2.534 | .941 | .335 | 0.868 | .012 | 0.361 | .550 | 0.841 | .005 | .271 | .604 | 0.872 | .004 |
| **SVF vegetables Total** | 14.50 | 4.161 | 13.45 | 2.762 | 12.85 | 2.996 | 13.80 | 3.928 | .680 | .412 | 0.928 | .009 | 0.004 | .950 | 0.950 | .000 | .991 | .323 | 0.826 | .013 |
| **Perseveration vegetables** | .55 | .826 | .40 | .598 | .15 | .366 | .95 | 1.146 | .180 | .672 | 0.945 | .002 | 3.384 | .070 | 0.708 | .043 | .001 | .978 | 0.978 | .000 |
| **Violation vegetables** | 1.05 | 1.638 | 2.00 | 1.556 | 1.80 | 1.824 | 1.05 | 1.317 | .079 | .779 | 0.945 | .001 | 0.079 | .779 | 0.863 | .001 | .226 | .636 | 0.887 | .003 |
| **SVF total** | 37.35 | 7.140 | 36.70 | 5.469 | 34.15 | 6.150 | 35.45 | 8.140 | 2.131 | .149 | 0.868 | .028 | 0.045 | .832 | 0.885 | .001 | .912 | .343 | 0.826 | .012 |
| **SVF total perseverations** | 1.25 | 1.446 | .95 | 1.356 | .70 | .657 | 1.45 | 1.395 | .008 | .929 | 0.947 | .000 | 0.645 | .425 | 0.841 | .009 | .268 | .606 | 0.872 | .004 |
| **SVF total violations** | 1.45 | 2.038 | 2.15 | 1.599 | 1.80 | 1.824 | 1.55 | 2.064 | .101 | .751 | 0.945 | .001 | 0.328 | .569 | 0.841 | .004 | 9.371 | **.003** | 0.080 | .111 |
| **Picture naming correct without cue** | 31.15 | 1.268 | 31.55 | .686 | 31.70 | .470 | 31.10 | 1.804 | .036 | .851 | 0.945 | .000 | 0.142 | .707 | 0.863 | .002 | .846 | .361 | 0.826 | .011 |
| **Picture naming correct with cue** | .075 | .2447 | .050 | .1539 | .075 | .1832 | .175 | .4667 | .921 | .340 | 0.868 | .012 | 0.332 | .566 | 0.841 | .004 | .112 | .739 | 0.890 | .001 |
| **Picture naming correct total** | 31.225 | 1.1295 | 31.600 | .6609 | 31.775 | .3796 | 31.275 | 1.4186 | .259 | .612 | 0.945 | .003 | 0.08 | .778 | 0.863 | .001 | 1.007 | .319 | 0.826 | .013 |
| **TMT A** | 37.05 | 18.211 | 49.40 | 18.560 | 40.85 | 17.373 | 36.25 | 17.541 | 1.345 | .250 | 0.868 | .018 | 0.924 | .339 | 0.841 | .012 | 1.092 | .299 | 0.826 | .014 |
| **TMT B** | 99.05 | 52.943 | 89.20 | 34.551 | 105.10 | 31.409 | 86.30 | 36.367 | .033 | .857 | 0.945 | .000 | 2.712 | .104 | 0.708 | .035 | 3.206 | .077 | 0.510 | .041 |
| **TMT B-A** | 62.00 | 38.003 | 39.80 | 38.506 | 64.25 | 25.876 | 50.05 | 23.401 | .824 | .367 | 0.884 | .011 | 6.985 | **.010** | 0.530 | .085 | 8.255 | **.005** | 0.088 | .099 |
